# Golgi fragmentation in Alzheimer's disease

**DOI:** 10.3389/fnins.2015.00340

**Published:** 2015-09-24

**Authors:** Gunjan Joshi, Michael E. Bekier, Yanzhuang Wang

**Affiliations:** ^1^Department of Molecular, Cellular and Developmental Biology, University of MichiganAnn Arbor, MI, USA; ^2^Department of Neurology, University of Michigan School of MedicineAnn Arbor, MI, USA

**Keywords:** Alzheimer's disease, amyloid precursor protein, amyloid beta, Golgi, GRASP65, phosphorylation

## Abstract

The Golgi apparatus is an essential cellular organelle for post-translational modifications, sorting, and trafficking of membrane and secretory proteins. Proper functionality of the Golgi requires the formation of its unique cisternal-stacking morphology. The Golgi structure is disrupted in a variety of neurodegenerative diseases, suggesting a common mechanism and contribution of Golgi defects in neurodegenerative disorders. A recent study on Alzheimer's disease (AD) revealed that phosphorylation of the Golgi stacking protein GRASP65 disrupts its function in Golgi structure formation, resulting in Golgi fragmentation. Inhibiting GRASP65 phosphorylation restores the Golgi morphology from Aβ-induced fragmentation and reduces Aβ production. Perturbing Golgi structure and function in neurons may directly impact trafficking, processing, and sorting of a variety of proteins essential for synaptic and dendritic integrity. Therefore, Golgi defects may ultimately promote the development of AD. In the current review, we focus on the cellular impact of impaired Golgi morphology and its potential relationship to AD disease development.

## Introduction

The Golgi apparatus is a membranous cellular organelle that mediates proper trafficking, post-translational processing, and sorting of membrane and secretory proteins. The Golgi is often localized to the perinuclear region of the cell, which depends on an intact microtubule network and a motor protein dynein. Golgi membranes form multilayer stacks that are laterally linked into a ribbon. Formation of stable multilayer stacks and ribbons appears to be essential for proper functioning of the Golgi (Rambourg et al., 1987; Ladinsky et al., 1999; Klumperman, 2011; Klute et al., 2011). The Golgi structure is controlled by Golgi matrix proteins, a group of peripheral and membrane proteins localized on the cytoplasmic surface of Golgi membranes, including the coiled-coil domain-containing golgins and the stacking proteins GRASP55/65 (Ramirez and Lowe, 2009; Xiang and Wang, 2011; Wong and Munro, 2014). Depletion of Golgi matrix proteins such as GRASP65 (Xiang et al., 2013; Veenendaal et al., 2014), GRASP55 (Feinstein and Linstedt, 2008; Xiang and Wang, 2010), GM130 (Puthenveedu et al., 2006), golgin-84 (Diao et al., 2003), and golgin-160 (Williams et al., 2006), or treatment of cells with pharmacological drugs such as Brefeldin A (BFA) (Klausner et al., 1992), results in an abnormal, fragmented Golgi morphology. Disruption of normal Golgi morphology directly impacts protein trafficking and processing. For example, breaking down the Golgi ribbon of mammalian cells by nocodazole treatment strongly inhibits intra-Golgi transport of large cargoes without altering the rate of transport of smaller cargoes (Lavieu et al., 2014), while inhibition of stack formation by knocking down GRASP55/65 by RNA interference (RNAi) accelerates protein trafficking, impairs proper glycosylation, and leads to missorting and delivery of lysosomal proteins to the extracellular space (Xiang et al., 2013). Thus, the structure of the Golgi is closely linked to vital cellular processes.

Golgi fragmentation has been observed in neurodegenerative diseases, including Alzheimer's (AD) (Stieber et al., 1996; Huse et al., 2002), Parkinson's (PD) (Mizuno et al., 2001), and Huntington's (HD) (Hilditch-Maguire et al., 2000) diseases and amyotrophic lateral sclerosis (ALS) (Mourelatos et al., 1996; Gonatas et al., 1998; Fujita and Okamoto, 2005). However, the molecular basis of Golgi fragmentation and its role in disease progression remain largely unexplored. Golgi defects may impact the trafficking and processing of many proteins essential for neuronal functions. Thus, morphological and subsequent functional impairments of the Golgi may contribute to the neurotoxicity associated with neurodegenerative diseases. Understanding the mechanisms that cause Golgi fragmentation and its downstream effects on neuronal functions are likely important for understanding the molecular basis of the diseases. Furthermore, targeting Golgi structural defects may represent a novel approach to treating or preventing related diseases with Golgi defects. In this review, we summarize the current literature on the cause and effect of Golgi fragmentation in AD, the leading cause of dementia in adults (Alzheimer's, 2015).

## Golgi and amyloid plaque formation

**Figure 1:**
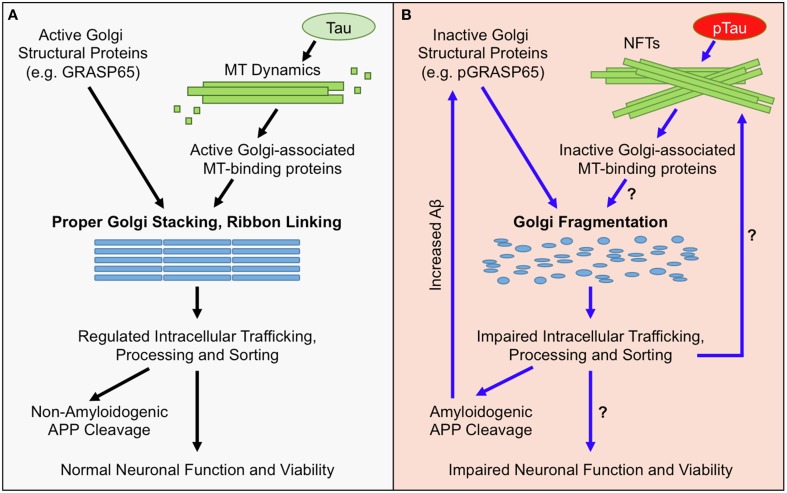
**Golgi Morphological and Functional Defects in AD**. **(A)** Under normal conditions, the structure of the Golgi is maintained by active Golgi structural proteins such as GRASP65 (non-phosphorylated) and an intact microtubule (MT) network. Maintaining the Golgi structure is essential for proper trafficking and processing of APP and its processing enzymes. The majority of APP undergoes non-amyloidogenic processing, and cell-surface proteins, lipids, and polysaccharides, which are essential for neuronal function, are properly sorted and transported. Together, these factors maintain neuronal functionality and viability. **(B)** In AD, the Golgi is fragmented due to inactivation of Golgi structural proteins, such as degradation or phosphorylation of GRASP65 (pGRASP65), or tau hyper phosphorylation (pTau) and NFT formation that disrupt MT dynamics and protein trafficking. Golgi fragmentation impairs trafficking, processing, and sorting of APP and APP-processing enzymes, which stimulates amyloidogenic APP cleavage and further inactivates GRASP65. Additionally, Golgi fragmentation is predicted to alter trafficking, processing, and sorting of proteins, lipids, and polysaccharides that are essential for neuronal function; which could ultimately promote neuronal dysfunction and/or cell death.

One neuropathological hallmark of AD is the formation of extracellular amyloid plaques by secreted amyloid beta (Aβ) peptides (Nelson et al., 2009), which is highly related to Golgi structure and function (Figure 1). Aβ is derived from the amyloid precursor protein (APP), a type I membrane protein that travels through the exocytic and endocytic pathways and undergoes sequential proteolysis by the action of β- and γ-secretases (Vassar et al., 1999). In neurons, APP is transported from the Golgi to many sub-cellular compartments (Haass et al., 1992), including the soma, dendrites and axons, through the exocytic and endocytic pathways. Despite the abundant literature demonstrating the critical role of endosomes in APP processing (for review see Suh and Checler, 2002; Small and Gandy, 2006), it has been indicated that the Golgi (in particular the *trans*-Golgi network) may be a site where Aβ is generated in the cell (Greenfield et al., 1999; Burgos et al., 2010; Choy et al., 2012). In addition, proper functioning of the Golgi is required for trafficking and maturation of both APP and its processing enzymes. For instance, the activity of the γ-secretase depends on the trafficking and maturation of nicastrin (Chung and Struhl, 2001) and other components of the γ-secretase complex through the Golgi (Herreman et al., 2003). Nicastrin is not catalytically active, but is important for the maturation and proper trafficking of the γ-secretase complex (Zhang et al., 2005). Nicastrin functions to stabilize presenilins (PSs), the catalytic subunit of the γ-secretase complex, and mediates PS trafficking to the cell surface by an unknown mechanism (Edbauer et al., 2002; Hu et al., 2002). Nicastrin also binds to the N-terminal domain of APP, and facilitates APP trafficking and cleavage (Yu et al., 2000; Kimberly et al., 2002). APP travels from the endoplasmic reticulum (ER) through the Golgi to the plasma membrane. The majority of APP localizes to the Golgi where it undergoes post-translational modifications and only a small fraction resides in the ER and the plasma membrane (Thinakaran and Koo, 2008). Finally, the α-secretatse ADAM10 is transported from dendritic Golgi outposts to synaptic membranes, a reaction modulated by the synapse-associated protein-97 (SAP97) (Saraceno et al., 2014). Thus, APP processing and Aβ production are intrinsically linked to the proper morphology and functionality of the Golgi.

## Golgi and tau pathology in AD

Another pathological distinction in AD is the formation of neurofibrillary tangles (NFTs) caused by precipitation of hyperphosphorylated tau protein, which is also related to Golgi structure and function (Figure 1) (Morishima-Kawashima and Ihara, 2002). Tau is an abundant protein that binds and stabilizes microtubules in neurons (Gong and Alonso Adel, 2013). Neurons have long protrusions such as axons; transport of membrane organelles and proteins into and from these protrusions highly depends on an intact microtubule network (Baas, 2002). Long microtubules emanate out of the centrosomes and function as the track for rapid transport of membranes by kinesin and dynein motors (Hancock, 2014). Membranes and proteins for synaptic vesicle formation and synaptic function are essentially all transported in this way (Gallant, 2000; Goldstein and Yang, 2000).

In AD, hyperphosphorylation of tau results in NFT formation, which impairs microtubule integrity and blocks membrane transport (Patrick et al., 1999; Avila, 2006; Baloyannis, 2014). Interestingly, the Golgi, whose formation relies on an intact microtubule network (Rios and Bornens, 2003), may also function as an important microtubule organization center (Lewis and Polleux, 2012; Ori-McKenney et al., 2012; Zhu and Kaverina, 2013). Multiple microtubule-binding proteins, such as γ-tubulin (Radulescu et al., 2011), CLASP (Liu et al., 2007; Miller et al., 2009), GMAP-210 (Infante et al., 1999; Roboti et al., 2015), and GM130 (Rivero et al., 2009), are localized on the Golgi membranes and modulate microtubule network formation and membrane trafficking in neurons (Rios, 2014; Tang et al., 2015) and astrocytes (Yoshiyama et al., 2003). It has been reported that tau interacts with Golgi membranes and mediates their association with microtubules (Farah et al., 2006). Overexpression of wild-type and mutant human tau proteins causes Golgi fragmentation in primary hippocampal neurons (Liazoghli et al., 2005), implying that overabundance of tau, and potentially NFT formation, precedes Golgi morphological defects. Additionally, Golgi fragmentation may affect tau phosphorylation (Jiang et al., 2014) and promote NFTs formation in AD (Grundke-Iqbal et al., 1986). Therefore, there is a close connection between Golgi morphology and function, microtubule organization, and tau pathology in AD.

## Golgi morphological defects in AD

Golgi morphological alterations have been observed in neurons of AD patients (Dal Canto, 1996; Stieber et al., 1996; Gonatas et al., 1998; Huse et al., 2002), which occur even before the formation of NFTs and neuritic plaques (Baloyannis, 2014). Golgi fragmentation has been recently confirmed in AD tissue culture and mouse models (Joshi et al., 2014). Expression of the Swedish mutant APP (APPswe) and the exon 9-deletion mutant PS1 (PS1ΔE9) in cells and mouse brain, or treatment of neurons with synthetic Aβ peptides, resulted in Golgi fragmentation. Under fluorescence microscopy, Golgi elements are unlinked and dispersed into the cytoplasm. Under electron microscopy (EM), the disconnected Golgi stacks exhibit reduced number of cisternae per stack, shorter cisternae, more vesicles surrounding each stack and a dilated Golgi structure compared with wild type cells and untreated neurons (Joshi et al., 2014). In this study, the direct cause of Golgi fragmentation is Aβ accumulation, as Aβ treatment causes Golgi fragmentation in cultured neurons and other cell types; this effect is reversible upon removal of Aβ from the tissue culture medium (Joshi et al., 2014). Golgi fragmentation has also been observed under physiological conditions, such as during migration, upon growth factor treatment, and in neurons with increased neuronal activity (Bisel et al., 2008; Thayer et al., 2013), suggesting that Golgi fragmentation may not be an immediate pathological response, but rather a compensatory reaction to allow fast transport of proteins to their final destinations when the cells are under stress. Golgi fragmentation, however, impacts protein glycosylation and sorting (Wang et al., 2008; Xiang et al., 2013), as we discuss below.

## Mechanisms of Golgi morphological defects in AD

The formation and maintenance of the perinuclear localization of the Golgi ribbon rely on an intact microtubule cytoskeleton emanating from the perinuclear centrosomes (Glick and Nakano, 2009; Yadav and Linstedt, 2011; Rios, 2014). The minus end-directed motor dynein associates with the Golgi and moves the membranes along the microtubules toward the centrosomes, leading to the concentration of the Golgi stacks in the pericentriolar region and formation of the Golgi ribbon (Rios and Bornens, 2003; Miller et al., 2009). The basic structural and functional unit of the Golgi is a stack of flattened cisternae. The exact mechanism for Golgi stack formation is not fully understood, but is believed to rely on Golgi structural proteins (Wang and Seemann, 2011; Xiang and Wang, 2011). Among the well characterized Golgi structural proteins, GRASP65 (Wang et al., 2003) and GRASP55 (Xiang and Wang, 2010) play essential roles in Golgi structure formation, whereas others, including GM130 (Lowe et al., 1998), golgin-160 (Hicks and Machamer, 2002), and p115 (Chiu et al., 2002), are more important for trafficking across the Golgi stack (Xiang and Wang, 2011). For example, GRASP65 forms oligomers that tether the cisternae into stacks (Wang et al., 2003; Tang et al., 2010; Lavieu et al., 2013) and ribbons (Puthenveedu et al., 2006). Recently a GRASP65 knockout mouse has been reported, with defects in *cis*-Golgi ribbon-linking and no apparent neurological phenotype (Veenendaal et al., 2014). One potential concern regarding this mouse strain is that some mRNA encoding exon 1–3 is still present (~20% of the mRNA level in wild-type mice). This truncated mRNA encodes a 115 aa N-terminal fragment of GRASP65. If translated (although not detected by available antibodies raised against the full length protein), this fragment would be sufficient for Golgi stacking, according to biochemical and structural studies (Tang et al., 2010; Truschel et al., 2011). However, a more recent structure (Feng et al., 2013) indicates that a longer fragment is needed for GRASP65 function in Golgi stack formation (Wang et al., 2003, 2005). The lack of a more prominent Golgi stacking and neurological phenotype may also be due to the complementation by GRASP55, the homolog of GRASP65 that shares some redundancy in stacking (Shorter et al., 1999; Lee et al., 2014).

**Figure 2:**
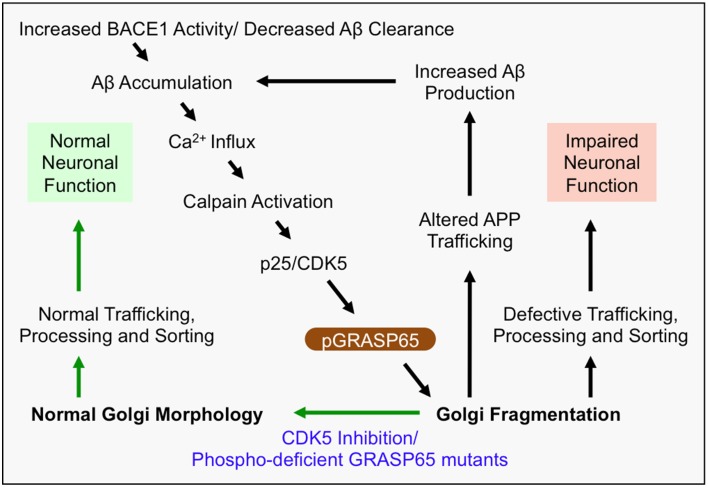
**Mechanism of Golgi defects in AD**. Increased BACE1 activity and/or decreased Aβ clearance from the extracellular space leads to the accumulation of Aβ. In turn, Aβ induces Ca^2+^ influx, which activates Calpain and induces cleavage of p35 to p25. Consequently, p25 activates CDK5, which in turn phosphorylates and inactivates the Golgi structural protein GRASP65 (i.e., pGRASP65). Inactivation of GRASP65 causes Golgi fragmentation, which alters trafficking of APP, and potentially the secretases, leading to increased Aβ production. This deleterious feedback loop (indicated by black arrows) would impair the integrity of the secretory pathway for sorting, trafficking and modifications of many essential proteins, which may compromise neuronal function, activate inflammatory responses, or cause neuronal cell death. Inhibition of CDK5 or expression of non-phosphorylatable GRASP65 mutants restores the normal Golgi morphology and reduces APP trafficking and Aβ production (indicated by green arrows). Therefore, rescue of the defective Golgi may delay AD development.

The mechanism of Golgi fragmentation in AD has not been well studied, but likely involves multiple mechanisms (Figure 1). One possibility is through the disruption of the microtubule network by tau precipitation and NFT formation (Liazoghli et al., 2005). Microtubule defects may affect both the central localization of the Golgi in the cell and ER-Golgi-plasma membrane trafficking that indirectly impacts the size and morphology of the Golgi (Fokin et al., 2014). Tau could also affect vesicle trafficking by inhibiting the binding of motor proteins such as kinesins to microtubules (Seitz et al., 2002). Another possibility is through modulation of Golgi structural proteins (Figure 2). Both mitotic phosphorylation and apoptotic cleavage of Golgi proteins results in Golgi fragmentation (Wang and Seemann, 2011). For instance, GRASP65 is phosphorylated by mitotic kinases Cdk1 and Polo-like kinase (Plk1) during mitosis (Wang et al., 2003) and cleaved by caspase-3 in apoptosis (Lane et al., 2002), both of which cause Golgi fragmentation (Tang et al., 2008; Wang and Seemann, 2011). In tissue culture and mouse models of AD, GRASP65 phosphorylation was implicated as a major cause of Golgi fragmentation (Joshi et al., 2014; Joshi and Wang, 2015). At the molecular level, Aβ accumulation triggers Ca^2+^ influx (Zempel et al., 2010), which activates Calpain, a protease known to increase the cleavage of p35 to p25 (Lee et al., 2000), p25 then activates Cdk5. It has been previously reported that p35 and Cdk5 are associated with Golgi membranes and regulate membrane traffic (Paglini et al., 2001). Subsequently, Cdk5 (also known to phosphorylate tau in AD) phosphorylates GRASP65, which negatively regulates GRASP65, leading to Golgi fragmentation. Consequently, Golgi fragmentation enhances APP trafficking and increases Aβ production (Joshi et al., 2014). Fragmentation of the Golgi was rapidly reversible by the use of Cdk5-specific inhibitors, or by expression of non-phosphorylatable GRASP proteins, both of which significantly reduced APP trafficking and Aβ production (Figure 2). In the same study, degradation of Golgi structural proteins was not detected (Joshi et al., 2014). These results suggest that Golgi fragmentation in AD, at least in the early stage, is caused by phosphorylation of Golgi structural proteins, an event that occurs in parallel with tau hyper-phosphorylation during the development of the disease. Overall, the causes of Golgi structural defects in AD are expected to be manifold and require further investigation to determine the precise mechanisms.

## Functional consequences of Golgi fragmentation in AD

Proper Golgi structure formation controls the sequence and speed of protein transport through the Golgi membranes for proper trafficking, maturation, sorting, and processing of not only APP, but also many neuronal proteins (Dries and Yu, 2008; Joshi and Wang, 2015). When BACE1 activity is increased or Aβ clearance is decreased, Aβ accumulation induces Golgi fragmentation through modification of GRASP65, and other Golgi structural proteins (Joshi et al., 2014; Joshi and Wang, 2015). Fragmentation of the Golgi, as one possible outcome, enhances vesicle budding from the Golgi membranes, accelerates protein trafficking, and impairs accurate glycosylation (Wang et al., 2008; Xiang et al., 2013), and thus increases Aβ production by enhancing amyloidogenic cleavage (Figure 2). APP and the β-secretase BACE1 are sorted by the Golgi into different compartments and the loss of the sorting function of the Golgi by fragmentation in AD results in defective trafficking of APP and BACE1, which promotes Aβ production (Tan and Evin, 2012; Das et al., 2013; Joshi et al., 2014). This deleterious feedback loop may impair the integrity of the secretory pathway for sorting, trafficking and modifications of many essential neuronal proteins. For example, many AD-related proteins that are essential for neuronal function, such as NMDA and AMPA receptors (Pérez-Otaño and Ehlers, 2005; Greger and Esteban, 2007; Shepherd and Huganir, 2007), and synaptic integrity proteins such as neurexins (Fairless et al., 2008; Reissner et al., 2013), are processed and trafficked through the Golgi. Thus, Golgi fragmentation might impair trafficking of membrane receptors and ion channels that regulate neuronal function and/or viability (Figure 1).

Long term Golgi defects might also result in a significant change in the composition of proteins, lipids, and polysaccharides at the cell surface (Figure 1). A change in the composition of cell-surface molecules could not only directly impair neuronal activity and synaptic integrity, but also potentially trigger an immune response against neurons with Golgi defects that leads to cell death. In addition, Golgi defects may impact microtubule organization, tau function, and cell polarity. The Golgi also functions as a storage reservoir for Ca^2+^ (Pinton et al., 1998; Griesbeck et al., 2001), a small molecule essential for neurotransmission. Ca^2+^ is a key regulator of membrane fusion (Pang and Sudhof, 2010), synaptic plasticity (Neher and Sakaba, 2008), and neurite growth (Tojima et al., 2011). Defects in calcium signaling has been observed in neurodegeneration (Wojda et al., 2008), and prolonged neuronal hyperexcitability and neuronal activity lead to Golgi fragmentation (Thayer et al., 2013). Taken together, Golgi defects may compromise neuron activity and survival through multiple pathways and serve as an important mechanism of pathogenesis in AD and other neurodegenerative diseases.

## Conclusion

The Golgi apparatus plays an essential role in trafficking and sorting of proteins that are vital for neuronal functions. The Golgi is fragmented in many neurodegenerative diseases, suggesting that Golgi defects may contribute to neurodegeneration. Golgi fragmentation likely involves multiple mechanisms. In a recent study on AD, Golgi fragmentation is caused by phosphorylation of the Golgi structural protein GRASP65 and Golgi fragmentation results in enhanced APP trafficking and Aβ production. Golgi fragmentation is predicted to impair intracellular trafficking of many proteins that are essential for neuronal function. Restoring Golgi morphological defects might be an attractive approach to treating, or even preventing, AD. Understanding the impact of Golgi pathology and determining the molecular mechanisms that cause Golgi fragmentation is expected to shed insight into novel therapeutic approaches for treating AD and potentially other neurodegenerative disorders.

### Conflict of interest statement

The authors declare that the research was conducted in the absence of any commercial or financial relationships that could be construed as a potential conflict of interest.
